# Tazemetostat, a Selective EZH2 Inhibitor, in Combination with Pembrolizumab for Recurrent or Metastatic Head and Neck Squamous Cell Carcinoma: A Phase 1 Trial

**DOI:** 10.3390/cancers17030437

**Published:** 2025-01-27

**Authors:** Peter J. Oppelt, Sidharth V. Puram, Jingxia Liu, Jessica C. Ley, Douglas Adkins

**Affiliations:** 1 Division of Medical Oncology, Washington University School of Medicine, 660 South Euclid, Campus Box 8056, St Louis, MO 63110, USA; poppelt@wustl.edu (P.J.O.); jcley@wustl.edu (J.C.L.); 2 Alvin J. Siteman Cancer Center, St Louis, MO 63110, USA; sidpuram@wustl.edu; 3 Division of Head and Neck Surgery, Department of Otolaryngology, Washington University School of Medicine, St Louis, MO 63110, USA; 4 Division of Public Health Sciences, Department of Surgery, Washington University School of Medicine, St Louis, MO 63110, USA; esther@wustl.edu

**Keywords:** tazemetostat, pembrolizumab, phase 1 trial, head and neck cancer, recurrent/metastatic squamous cell carcinoma

## Abstract

**Objectives:** The primary aim of this phase 1 trial is to establish the recommended phase 2 dose (RP2D) of tazemetostat given with a fixed dose of pembrolizumab in patients with recurrent or metastatic head and neck squamous cell carcinomas (RM-HNSCCs). **Methods:** A 3 + 3 dose-escalation phase 1 design was used to assess three dose-levels of tazemetostat (400, 600, and 800 mg orally, twice daily) with pembrolizumab (200 mg intravenously). Cycle 1 was 35 days (tazemetostat days 1–35; pembrolizumab day 15). Subsequent cycles were 21 days (tazemetostat days 1–21; pembrolizumab day 1). Dose-limiting toxicity (DLT), assessed during cycle 1, was defined as study-drug-related grade 4 neutropenia or thrombocytopenia, grade 3 febrile neutropenia, or grade 3–4 non-hematologic adverse events (AEs). Patients had to have completed cycle 1 to be evaluable for the DLT assessment; otherwise, an equal number of additional patients were enrolled. The RP2D was defined as the highest dose level in which zero of three or less than or equal to one of six patients experienced a DLT. **Results:** Twelve patients were enrolled: three on 400 mg, three on 600 mg, and six on the 800 mg dose level of tazemetostat. Three patients on the 800 mg dose level did not complete cycle 1 and were not evaluable for DLT. In the other nine patients, DLTs did not occur during cycle 1. In all 12 patients, the most common AEs included anemia (10 patients), fatigue (eight), and hyponatremia (seven). **Conclusions:** Among the patients with RM-HNSCCs, the RP2D of tazemetostat was 800 mg and administered twice daily when given with pembrolizumab.

## Simple Summary

Pembrolizumab yields long-term benefits to a fraction of patients with recurrent head and neck cancer. Laboratory studies have shown that tumor control is improved with the addition of tazemetostat to pembrolizumab. This drug combination has not been tested in patients with recurrent head and neck cancer. In this clinical trial, we assessed the safety of three dose levels of tazemetostat given with a fixed dose of pembrolizumab in patients with recurrent head and neck cancer. We found that the 800 mg dose level of tazemetostat given with pembrolizumab was safe and tolerable. The most common side effects were a low red blood cell count and fatigue.

## 1. Introduction

Over 800,000 cases of head and neck squamous cell carcinomas (HNSCCs) are diagnosed each year [1]. Following curative-intent surgery and/or radiation-based therapies, half of these cases develop incurable, recurrent or metastatic (RM) diseases. First-line palliative systemic therapy includes the PD-1 inhibitor pembrolizumab given alone or with platinum-based chemotherapy [2]. The objective response rate (ORR) with pembrolizumab is low (17%); however, tumor responses are durable (median duration of response [DoR] 22·6 months). Chemotherapy added to pembrolizumab results in more frequent, but substantially less durable, tumor responses (ORR 36%; median DoR 6·7 months). Although some patients achieve a durable tumor response with pembrolizumab-based therapies, 80% of patients experience disease progression or death within 12 months. Subsequent therapies offer limited benefit. Novel strategies are necessary to improve the efficacy of immunotherapy for patients with RM-HNSCCs.

The mechanisms of resistance to the immune response in HNSCCs were recently reviewed [3]. Specific mechanisms of immune escape include decreased expression or mutation of HLA class I molecules and of antigen processing machinery, increased expression of immune inhibitory checkpoints on T lymphocytes, increased expression of PD-L1 on tumor cells, and production of inhibitory cytokines by tumor cells.

The chromatin modifier enhancer of zeste homolog 2 (EZH2) protein, a subunit of the polycomb repressor complex 2 (PRC2), catalyzes histone H3 lysine 27 trimethylation (H3K27me3) of target gene promotors and silences the transcription of several cancer-related genes via chromatin reorganization [4]. EZH2 represses the transcription of genes involved in the cell cycle arrest and terminal differentiation. EZH2 activity promotes cell proliferation, migration, and invasion [5]. EZH2 activity is opposed by the switch/sucrose non-fermentable (SWI/SNF) chromatin-remodeling multiprotein complex. In cancer, alterations of EZH2 include gain- or loss-of-function gene mutations, overexpression of the EZH2 protein, and mutations of the H3K27 demethylase gene UTX or the SWI/SNF (INI1, SMARCA4, or SMARCA2) chromatin-remodeling complex.

EZH2 overexpression is common in HNSCCs [6,7]. EZH2 mRNA levels are three-fold higher in HNSCCs compared to adjacent normal tissues [6]. A high expression of EZH2 correlates with DNMT3A overexpression and methylation of several tumor suppressor genes. EZH2 siRNA has been shown to downregulate EZH2 mRNA and protein in an EZH2-expressing HNSCC cell line (FaDu) and reduce colony formation [7]. EZH2 inhibitors decrease H3K27me3 in human papillomavirus (HPV)-positive and HPV-negative cell lines [8]. Targeted selective inhibition of EZH2 with tazemetostat upregulates the expression of HLA class I molecules in anti-PD-1-resistant HNSCC cell lines and mouse models and increases antigen-specific CD8+ T cell proliferation, IFN-gamma production, CXCL10 expression, and tumor cell cytotoxicity [9]. In an anti-PD-1-resistant HNSCC model, the combination of tazemetostat and anti-PD-1 suppresses tumor growth [9].

Tazemetostat monotherapy is safe to administer to patients with cancer. In a phase 1 trial, treatment-related adverse events (TRAEs) include asthenia, anemia, anorexia, muscle spasms, nausea, and vomiting [10]. One case of dose-limiting toxicity (DLT) has occurred: grade 4 thrombocytopenia at the highest dose (1600 mg bid). The recommended phase 2 dose (RP2D) of tazemetostat has been determined to be 800 mg orally twice daily, based on AEs, pharmacokinetics, on-target pharmacodynamics, and antitumor activity. Objective tumor responses have occurred in some patients with B-cell non-Hodgkin lymphoma and advanced solid tumors. To our knowledge, only one published study investigated tazemetostat in combination with an immune checkpoint inhibitor. In a phase 1b trial of patients with lymphoma, the RP2D of tazemetostat was 800 mg orally twice daily when given with the PD-L1 inhibitor atezolizumab [11].

Patients with RM-HNSCCs have different co-morbidities and residual treatment- and disease-related toxicity burdens compared to patients with lymphomas. Therefore, the RP2D of tazemetostat given in combination with an immune checkpoint inhibitor may be different in patients with RM-HNSCCs. In this phase 1 trial, we hypothesize that administration of tazemetostat in combination with pembrolizumab to patients with RM-HNSCCs would be safe and tolerable. The primary aim of the trial is to establish the RP2D of tazemetostat given with a fixed dose of pembrolizumab.

## 2. Materials and Methods

### 2.1. Study Design and Participants

This phase 1 trial assessed the safety and tolerability of tazemetostat given with pembrolizumab in patients with RM-HNSCCs. The trial was conducted at a single center. All patients provided written informed consent. The Washington University Institutional Review Board approved the study protocol (NCT04624113). In a 3 + 3 dose-escalation phase 1 design, the RP2D of tazemetostat was determined when given with a fixed dose of pembrolizumab. A planned single-arm, phase 2 component of this trial was not performed due to discontinuation of funding by Epizyme, an Ipsen Company (Cambridge, MA, USA) for reasons beyond the study itself. Only details of the phase 1 trial are presented here.

Eligible patients were 18 years of age or older with incurable RM-HNSCCs originating in the oral cavity, oropharynx, larynx, hypopharynx, nasopharynx, or skin. Incurable RM-HNSCCs were defined as local or regional recurrences, distant metastases, or diseases not amenable to (or patient declined) potentially curative surgery or radiation therapy. Patients were permitted to have measurable or evaluable disease and any number of lines of prior therapy for RM disease. Other inclusion criteria included an Eastern Cooperative Oncology Group (ECOG) performance status of 0–1 and adequate marrow and organ function (absolute neutrophil count [ANC] ≥ 750/mcl; hemoglobin > 9.0 g/dL; platelets ≥ 75,000/mcl; total bilirubin ≤ 1.5 mg/dL; AST, ALT ≤ 3× ULN; creatinine < 1.5× ULN or creatinine clearance ≥ 50 mL/min/1.73 m^2^). Key exclusion criteria included an inability to swallow tablets, active central nervous system metastases, corticosteroid or other immunosuppressive therapies, or active autoimmune disease. All study participants provided written informed consent to participate. The Washington University Human Research Protection Office approved the protocol and subsequent amendments. The trial was conducted in accordance with good clinical practice guidelines. The Washington University quality assurance and scientific monitoring committee performed independent data monitoring of the trial.

### 2.2. Procedures

Before the treatment, patients underwent a physical examination, a complete blood count, a metabolic panel, a urinalysis, free thyroxine (T4), thyroid stimulating hormone (TSH), a coagulation panel, and computerized tomography (CT) scans. Three dose levels of tazemetostat (400, 600, and 800 mg orally twice daily) were assessed along with a fixed dose (200 mg intravenously [IV]) of pembrolizumab. Cycle 1 was 35 days, with tazemetostat given on days 1–35 and pembrolizumab on day 15. Subsequent cycles were 21 days, with tazemetostat given on days 1–21 and pembrolizumab on day 1. The starting dose level of tazemetostat was 400 mg twice daily, taken with or without food, and was escalated in subsequent cohorts to 600 mg twice daily and then to 800 mg twice daily if pre-specified DLT criteria did not occur at a given dose level.

DLT, assessed during cycle 1, was defined as study-drug-related grade 4 neutropenia (for >7 days) or thrombocytopenia, grade 3 febrile (temperature ≥ 38.5 °C) neutropenia, or grades 3–4 non-hematologic AEs (except for nausea, vomiting, or anorexia). Each dose level cohort included a minimum of three patients. Patients had to have completed cycle 1 to be evaluable for the DLT assessment; otherwise, an equal number of additional patients were enrolled at that dose level. If zero of three evaluable patients experienced a DLT at a given dose level, escalation to the next dose level occurred. If one of three patients experienced a DLT at a given dose level, three additional patients were enrolled at the same dose level. If more than or equal to two of three or more than or equal to two of six patients experienced a DLT at the starting dose level, the study was to be halted. If more than or equal to two of three or more than or equal to two of six patients experienced a DLT at the second or third dose level, the subsequent patients were to be enrolled into the next lower dose level, up to a maximum of six patients. The RP2D was defined as the highest dose level in which zero of three or more than or equal to one of six evaluable patients experienced a DLT.

The treatment continued until disease progression, unacceptable toxicity, or discontinuation for any other reason. After treatment discontinuation, patients were followed to monitor for AEs (for 28 days) and survival.

Guidelines for dose delays and modifications of tazemetostat included: (1) grade 4 anemia: delay until recovery to ≤grade 1 or to baseline, then resume at the same dose, (2) ANC < 1000/mcl: delay until ANC ≥ 1000/mcl or to baseline, then resume at the same dose, with plans to reduce the dose for the second or third occurrence and discontinue it for the fourth occurrence, (3) platelets < 50,000/mcl: delay until recovery to ≥75,000/mcl or to baseline, then resume at a reduced dose for the first and second occurrence, with a plan to discontinue it for the occurrence, (4) grade 3 non-hematologic AEs: delay until recovery to ≤grade 1 or to baseline, then resume at a reduced dose for the first and second occurrence, with plans to discontinue it for the third occurrence, and (5) grade 4 non-hematologic AEs: delay until recovery to ≤grade 1 or to baseline, then resume at a reduced dose, with a plan to discontinue it for the second occurrence. Up to two dose level reductions of tazemetostat were permitted and included dose level 1 (75% of the prior dose) and dose level 2 (50% of the prior dose). Dose re-escalations following dose level reductions were not permitted. Subjects were removed from the trial for dose delays of tazemetostat exceeding 21 days. Guidelines for dose delays or holds of pembrolizumab were based on the drug’s package insert document. Dose delays or holds, but not dose reductions, of pembrolizumab were permitted. Patients could continue tazemetostat if pembrolizumab was held or discontinued. Patients could continue pembrolizumab if tazemetostat was held.

Tumor responses were assessed by independent radiology reviewers using the Response Evaluation Criteria in Solid Tumors version 1.1 (RECIST v1.1), as well as the modified iRECIST guidelines, with CT scans performed after every two cycles of therapy [12,13]. AEs were graded using the National Cancer Institute Common Toxicity Criteria for Adverse Events (NCI-CTCAE) version 5.0. The investigator judged the relationship of the AEs to the study drugs. A physical examination, complete blood count, and metabolic panel were performed on days 1 and 15 of cycle 1, and then on day 1 of each subsequent cycle. Free T4 and TSH were assessed after every four cycles.

PD-L1 expression was assessed by immunohistochemistry (IHC) using the DAKO 22C3 clone, and the combined positive score (CPS: the number of positively staining tumor and immune cells relative to the total number of tumor cells) was reported with CPS 0 being negative and CPS ≥ 1 being positive. For oropharynx SCCs, expression of p16, a surrogate biomarker of the HPV, was assessed by IHC. Tumors with strong (≥70%) and diffuse staining for p16 were scored as positive; otherwise, tumors were scored as negative. The HPV status was defined as HPV-positive (p16 positive oropharynx tumors) vs HPV-negative (p16 negative oropharynx tumors and tumors of all other primary sites) HNSCCs. Cases of p16 positive oropharynx SCCs with in situ hybridization or polymerase chain reactions were scored as HPV-negative. EZH2 expression levels were not assessed.

### 2.3. Outcomes and the Statistical Analysis Plan

The primary endpoints of phase 1 were the safety and tolerability of tazemetostat given in combination with pembrolizumab, assessed by DLTs that occurred during cycle 1. Secondary endpoints were AEs across all cycles of therapy and tumor responses. Patient compliance with taking tazemetostat was assessed using pill counts, cross-checked with a patient medication diary.

AEs were summarized by type and grade. All patients who received at least one dose of the study treatment were evaluable for AEs. Patients with a measurable disease who received at least one dose of the study treatment and had undergone at least one response assessment were evaluable for the response.

### 2.4. Role of the Funding Source

Epizyme, an Ipsen Company, funded the study. The funder had no role in the study design or writing of the report. The authors performed the data analysis and interpretation. All authors had full access to all the data in the study. The corresponding author had the final responsibility for the decision to submit for publication.

The study data were collected and managed using REDCap version 11 (research electronic data capture) electronic data capture tools hosted by the Washington University [14,15]. REDCap is a secure, web-based software platform designed to support data capture for research studies, providing (1) an intuitive interface for validated data capture; (2) audit trails for tracking data manipulation and export procedures; (3) automated export procedures for seamless data downloads to common statistical packages; and (4) procedures for data integration and interoperability with external sources.

## 3. Results

### 3.1. Patient Characteristics

Twelve patients signed the consent form for the study and were eligible and enrolled into the trial. The patient and tumor characteristics are shown in Table 1.

The median age of the patients was 63 years (range: 41–79). Eleven tumors were HPV-negative and one was HPV-positive. The PD-L1 CPS status of the tumors was positive in 10 patients. Eight of the 12 patients had received prior anti-PD1 therapy to treat RM diseases. The best tumor responses to prior anti-PD1 therapy were complete (1) or partial responses (2), stable disease (2) or progressive disease (3).

### 3.2. Treatment

Twelve patients were treated with at least one dose of the study drug. Three patients were treated with the 400 mg, three with the 600 mg, and six with the 800 mg dose level of tazemetostat. Three patients (all on the 800 mg dose level) did not complete cycle 1 due to early disease progression. Nine patients completed cycle 1. In these nine patients, the study drugs were subsequently discontinued after cycle 1 in one patient, during or after cycle 2 in three, after cycle 3 in two, after cycle 4 in one, after cycle 5 in one, and after cycle 8 in one. Dose holds or discontinuations of tazemetostat due to AEs occurred in one patient, and of pembrolizumab in zero patients.

By the last follow-up, the study drug treatment had been discontinued in all 12 patients due to disease progression (10 patients), the interim inability to swallow tazemetostat (one), or patient decision to withdraw from the trial (one).

### 3.3. DLT Assessment and RP2D Determination

Nine patients (three on each dose level) completed cycle 1 of the therapy and were evaluable for the DLT endpoint. The AEs that occurred in these nine patients are shown in Table 2. DLT events did not occur during cycle 1 in these patients. Thus, the RP2D of tazemetostat was determined to be 800 mg orally twice daily when given with a fixed dose (200 mg IV) of pembrolizumab.

### 3.4. Adverse Events Across All Cycles of the Therapy

AEs that occurred in all 12 patients across all the cycles of the therapy are also shown in Table 2. The most common AEs included anemia (10 patients), fatigue (eight), hyponatremia (seven), elevated alkaline phosphatase (seven), and dry skin (five). Notably, pre-treatment lymphopenia was present in 11 patients. Grade 4 or 5 treatment-related AEs (TRAE) did not occur in these 12 patients. Grade 3 TRAEs occurred in two patients; both patients experienced grade 3 anemia at the 800 mg dose level. Two grade 4 non-TRAEs occurred, both related to the cancer: tracheal obstruction (1) and acute respiratory failure due to aspiration pneumonia (1). Immune-related (ir) AEs requiring corticosteroids did not occur. One case of hypothyroidism requiring thyroid hormone supplementation did occur. No other irAEs occurred.

### 3.5. Tumor Response and Current Patient Status

Objective tumor responses did not occur in the 12 patients on the trial. The best tumor responses were a stable disease in five patients (two at the 400 mg, two at the 600 mg, and one at the 800 mg dose level of tazemetostat) and a progressive disease in seven patients. The median duration of the stable disease was 2.1 months (range 0.8–4.4). The median follow-up was 7 months (range 1.5–31.1). By the last follow-up, 10 patients had expired (all due to disease progression).

## 4. Discussion

Among the patients with RM-HNSCCs, this phase 1 trial demonstrated that the RP2D of tazemetostat was 800 mg orally twice daily when given with a fixed dose (200 mg IV) of pembrolizumab. DLTs did not occur across the three dose levels of tazemetostat. When tazemetostat is given as a single agent, the recommended dose is 800 mg orally twice daily. We show that tazemetostat in combination with pembrolizumab did not harm the delivery of either drug. Dose modifications, holds, or discontinuations of either study drug due to AEs occurred in only one patient. The explanation for this observation likely relates to the non-overlapping AE profiles of these two drugs.

In this phase 1 trial, the AEs that occurred across all cycles of the therapy were generally mild to moderate in severity and tolerability, consistent with the known AEs associated with each drug or with the AEs seen in patients with previously treated RM-HNSCCs. The most common AEs observed included anemia, fatigue, hyponatremia, elevated alkaline phosphatase, and dry skin. Most AEs were grade 1–2 in severity. Grade 3 TRAEs occurred in two patients (anemia in both). Notably, anemia occurred in 10 of the 12 patients (grade 3 in three patients). Cases of grade 3 anemia were managed with transfusion support and/or closer monitoring, as needed. No cases of hematologic malignancies occurred. Anemia is a common TRAE with the tazemetostat monotherapy, occurring in 50% of patients (grade 3 in 8%), but is also common among patients with RM-HNSCCs. Clinicians should carefully monitor for the development or worsening of anemia in patients given tazemetostat. Grade 4 or 5 TRAEs did not occur in these patients.

It is possible that the immunologic effects of tazemetostat observed in pre-clinical models could enhance the frequency, severity, and pattern of irAEs due to pembrolizumab. We did not find this to be true in this phase 1 trial; however, the sample size was small. Phase 2 trials with larger sample sizes will be important to clarify this concern.

To our knowledge, only one other published study investigated tazemetostat in combination with an immune checkpoint inhibitor. In a phase 1b trial, tazemetostat was combined with a fixed dose (1200 mg IV every three weeks) of atezolizumab, a PD-L1 inhibitor, in 43 patients with lymphomas [11]. The RP2D of tazemetostat was 800 mg orally twice daily when given with atezolizumab. No DLT events occurred at this dose level of tazemetostat. The most common AEs reported were anemia, fatigue, and nausea. The frequency, severity, and pattern of irAEs with this drug combination did not appear to be different from that observed with single-agent atezolizumab.

Pre-treatment lymphopenia was present in nearly all patients in this phase 1 trial, an observation recognized by others to be common in patients with RM-HNSCCs [16]. Baseline lymphopenia impairs the benefit of PD-1 inhibitors in patients with RM-HNSCCs and could be a barrier to strategies that seek to improve the efficacy of pembrolizumab [17].

Objective tumor responses did not occur in this trial. However, this phase 1 trial was not designed to formally test a hypothesis of an efficacy endpoint. Reasons why tumor responses were not observed may include the small sample size, the absence of biomarker selection, and the enrollment of patients with heavily pretreated diseases resistant to multiple therapeutics including PD-1 inhibitors in most cases. Also, the ORR with PD-1 inhibitor alone in patients with platinum-pretreated and untreated RM-HNSCCs was low (13–14% and 17%, respectively), such that a trial with a large sample size may be required to demonstrate tumor responses with tazemetostat and pembrolizumab [2,18,19]. Five of the 12 patients experienced a stable disease as the best response to tazemetostat and pembrolizumab; however, the duration of the stable disease was brief, indicating little impact on the pace of disease growth in these cases. Additional trials will be needed to determine if tazemetostat enhances the efficacy of pembrolizumab in patients with RM-HNSCCs. Pre-clinical data showed that cisplatin induced EZH2 expression and that EZH2 inhibition enhanced cisplatin-induced cell death, implying that combinations of tazemetostat and cisplatin with or without pembrolizumab may be worthy of evaluation in patients with RM-HNSCCs [20].

Although the activity of tazemetostat in carcinomas is unclear, tumor responses with single-agent tazemetostat have been reported in patients with other cancer types. The tumor response rate of tazemetostat in epitheliod sarcomas with loss of INI1/SMARCB1 was 15% [21]. In relapsed or refractory follicular lymphomas, the tumor response rate with tazemetostat was 34% in EZH2 wild-type diseases and 69% in EZH2 mutant diseases [22]. Some of these responses were durable. The tumor response rate with tazemetostat in combination with atezolizumab was 16% in patients with biomarker-unselected relapsed or refractory diffuse large B cell lymphoma [11].

Our study had limitations. The AE profile observed with tazemetostat and pembrolizumab in this small phase 1 trial may be different to that observed in studies with larger sample sizes. A planned single-arm phase 2 component of this trial was not performed due to discontinuation of funding. The phase 2 component of the trial was designed to test the primary hypothesis that tazemetostat and pembrolizumab would result in objective tumor responses in patients with anti-PD-1-resistant, PD-L1 positive RM-HNSCCs. However, objective tumor responses were not observed in the phase 1 component of the trial.

## 5. Conclusions

Among the patients with RM-HNSCCs, the RP2D of tazemetostat was 800 mg orally twice daily when given with pembrolizumab.

## Figures and Tables

**Table 1:** Patient and tumor characteristics.

Characteristic	Phase 1 (n = 12)
Age (years), median	63 (Range: 41–79)
Sex	
Male	11
Female	1
Race	
Caucasian	8
African American	3
Asian	1
ECOG Performance Status ^a^	
0	8
1	4
Smoking History	
Yes	7
No	5
HPV ^b^ Status and Primary Site	
HPV positive (oropharynx only)	1
HPV negative	11
Larynx	4
Oral cavity	3
Oropharynx	2
Skin	1
Nasopharynx	1
PD-L1	
≥1	10
1–19	7
≥20	3
0	1
Unknown	1
Lines of Prior Treatment for RM-HNSCCs	
1–2	6
3+	6
Prior anti-PD1 therapy for RM-HNSCCs	
Yes	8
<6 months ago	5
≥6 months ago	3
No	4

^a^ Eastern Cooperative Oncology Group. ^b^ Human Papillomavirus.

**Table 2:** Adverse events that occurred at each of the three dose levels of tazemetostat across all cycles of the therapy.

Adverse Event	400 mg Dose Level ^d^ (n = 3)			600 mg Dose Level ^d^ (n = 3)			800 mg Dose Level ^d^, Evaluable for DLT (n = 3)			800 mg Dose Level ^d^, Non-Evaluable for DLT (n = 3)			All Patients (n = 12)		
	Grade			Grade			Grade			Grade			Grade		
	1–2	3	4	1–2	3	4	1–2	3	4	1–2	3	4	1–2	3	4
Alanine aminotransferase increase										2	1 ^b^		2	1 ^b^	
Alkaline phosphatase increase	3			1						3			7		
Anemia	2	1 ^a^		3				1 ^c^		2	1 ^c^		7	3 ^a,c^	
Anorexia	1			1			2						4		
Aspartate aminotransferase increase	1									2			3		
Confusion	1												1		
Creatinine increase	1												1		
Dehydration	1												1		
Delirium		1 ^a^												1 ^a^	
Diarrhea				1									1		
Dizziness	2												2		
Dry skin	3			2									5		
Dyspnea	2			1									3		
Edema limbs				1									1		
Fatigue	1	1 ^a^		3						3			7	1 ^a^	
Generalized muscle weakness				1									1		
Headache	2						1						3		
Hoarseness	1			1									2		
Hypercalcemia										1			1		
Hyperkalemia	1												1		
Hypertension	3			1	1 ^a^								4	1 ^a^	
Hypoalbuminemia	2			1			1			1			5		
Hypocalcemia		1 ^a^		1									1	1 ^a^	
Hypokalemia	1												1		
Hyponatremia	2			2			2			1			7		
Hypophosphatemia	2												2		
Hypotension	1			1									2		
Hypoxia	1												1		
Hypothyroidism	1												1		
Lung infection		1 ^a^			2 ^a^									3 ^a^	
Lymphedema	1												1		
Lymphocyte count decrease					1 ^a^			1 ^a^						2 ^a^	
Myalgia	1												1		
Non-cardiac chest pain	1												1		
Platelet count decrease										1			1		
Pleural effusion					1 ^a^									1 ^a^	
Pneumonitis				1									1		
Proteinuria							1						1		
Pruritus	2			1									3		
Respiratory failure			1 ^a^												1 ^a^
Sinus tachycardia	2			1									3		
Syncope		1 ^a^												1 ^a^	
Thromboembolic event	1												1		
Tracheal obstruction			1 ^a^												1 ^a^
Tumor pain		1 ^a^		1			1			1	1 ^a^		3	2 ^a^	
Weight loss	1				1 ^b^		1						2	1 ^b^	
White blood cell decrease							1						1		

Relationship of grade 3 or 4 AEs with the study drugs included: ^a^ unrelated, ^b^ unlikely related, ^c^ possibly related. ^d^ tazemetostat dose level.

## Data Availability

The individual participant data that underlie the results reported in this article, after de-identification (text, tables, figures, and appendices), and the study protocol will be shared, beginning 9 months and ending 24 months following article publication, with investigators whose proposed use of the data has been approved by an independent review committee (“learned intermediary”) identified for this purpose. Types of acceptable analyses include approved proposal(s) or individual participant data for meta-analyses. Proposals may be submitted up to 24 months following article publication. Information regarding submitting proposals and accessing the data may be submitted to jcley@wustl.edu.

## References

[1] Fitzmaurice C., Akinyemiju T.F., Al Lami F.H., Alam T., Alizadeh-Navaei R., Allen C., Alsharif U., Alvis-Guzman N., Amini E., Anderson B.O. (2018). Global Burden of Disease Cancer Collaboration. Global, Regional, and National Cancer Incidence, Mortality, Years of Life Lost, Years Lived With Disability, and Disability-Adjusted Life-Years for 29 Cancer Groups, 1990 to 2016: A Systematic Analysis for the Global Burden of Disease Study. JAMA Oncol..

[2] Burtness B., Harrington K.J., Greil R., Soulieres D., Tahara M., de Castro G., Psyrri A., Baste N., Neupane P., Bratland A. (2019). Pembrolizumab alone or with chemotherapy versus cetuximab with chemotherapy for recurrent or metastatic squamous cell carcinoma of the head and neck (KEYNOTE-048): A randomised, open-label, phase 3 study. Lancet.

[3] Kim K.H., Roberts C.W.M. (2016). Targeting EZH2 in cancer. Nat. Med..

[4] Jiao L., Liu X. (2015). Structural basis of histone H3K27 trimethylation by an active polycomb repressive complex 2. Science.

[5] Yan K.S., Lin C.Y., Liao T.W., Peng C.M., Lee S.C., Liu Y.J., Chan W.P., Chou R.-H. (2017). EZH2 in cancer progression and potential application in cancer therapy: A friend or foe?. Int. J. Mol. Sci..

[6] Kidani K., Osaki M., Tamura T., Yamaga K., Shomori K., Ryoke K., Ito H. (2009). High expression of EZH2 is associated with tumor proliferation and prognosis in human oral squamous cell carcinomas. Oral. Oncol..

[7] Mochizuki D., Misawa Y., Kawasaki H., Imai A., Endo S., Mima M., Yamada S., Nakagawa T., Kanazawa T., Misawa K. (2018). Aberrant epigenetic regulation in head and neck cancer due to distinct EZH2 overexpression and DNA hypermethylation. Int. J. Mol. Sci..

[8] Lindsay C.D., Kostiuk M.A., Harris J., O’Connell D.A., Seikaly H., Biron V.L. (2017). Efficacy of EZH2 inhibitory drugs in human papillomavirus-positive and human papillomavirus-negative oropharyngeal squamous cell carcinomas. Clin. Epigenetics.

[9] Zhou L., Mudianto T., Ma X., Riley R., Uppaluri R. (2020). Targeting EZH2 enhances antigen presentation, antitumor immunity, and circumvents anti-PD-1 resistance in head and neck cancer. Clin. Can. Res..

[10] Italiano A., Soria J.-C., Toulmonde M., Michot J.-M., Lucchesi C., Varga A., Coindre J.-M., Blakemore S.J., Clawson A., Suttle B. (2018). Tazemetostat, an EZH2 inhibitor, in relapsed or refractory B-cell non-Hodgkin lymphoma and advanced solid tumours: A first-in-human, open-label, phase 1 study. Lancet Oncol..

[11] Palomba M.L., Cartron G., Popplewell L., Ribrag V., Westin J., Huw L.-Y., Agarwal S., Shivhare M., Hong W.-J., Raval A. (2022). Combination of Atezolizumab and Tazemetostat in patients with relapsed/refractory diffuse large B-cell lymphoma: Results from a phase Ib study. Clin. Lymphoma Myeloma Leuk..

[12] Eisenhauer E.A., Therasse P., Bogaerts J., Schwartz L.H., Sargent D., Ford R., Dancey J., Arbuck S., Gwyther S., Mooney M. (2009). New response evaluation criteria in solid tumours: Revised RECIST guideline (version 1.1). Eur. J. Cancer.

[13] Seymour L., Bogaerts J., Perrone A., Ford R., Dancey J., Arbuck S., Gwyther S., Mooney M., Lin N.U., Litière S. (2017). iRECIST: Guidelines for response criteria for use in trials testing immunotherapeutics. Lancet Oncol..

[14] Harris P.A., Taylor R., Thielke R., Payne J., Gonzalez N., Conde J.G. (2009). Research electronic data capture (REDCap)—A metadata-driven methodology and workflow process for providing translational research informatics support. J. Biomed. Inform..

[15] Harris P.A., Taylor R., Minor B.L., Elliott V., Fernandez M., O’Neal L., McLeod L., Delacqua G., Delacqua F., Kirby J. (2019). The REDCap consortium: Building an international community of software partners. J. Biomed. Inform..

[16] Yasumatsu R., Wakasaki T., Hashimoto K., Nakashima K., Manako T., Taura M., Matsuo M., Nakagawa T. (2019). Monitoring the neutrophil-to-lymphocyte ratio may be useful for predicting the anticancer effect of nivolumab in recurrent or metastatic head and neck cancer. Head. Neck.

[17] Ho W.J., Yarchoan M., Hopkins A., Mehra R., Grossman S., Kang H. (2018). Association between pretreatment lymphocyte count and response to PD1 inhibitors in head and neck squamous cell carcinomas. J. Immunother. Cancer.

[18] Ferris R.L., Blumenschein G., Fayette J., Guigay J., Colevas A.D., Licitra L., Harrington K., Kasper S., Vokes E.E., Even C. (2016). Nivolumab for recurrent squamous-cell carcinoma of the head and neck. N. Engl. J. Med..

[19] Cohen E.E.W., Soulieres D., Le Tourneau C., Dinis J., Licitra L., Ahn M.-J., Soria A., Machiels J.-P., Mach N., Mehra R. (2018). Pembrolizumab versus methotrexate, docetaxel, or cetuximab for recurrent or metastatic head-and-neck squamous cell carcinoma (KEYNOTE-040): A randomised, open-label, phase 3 study. Lancet.

[20] Samaržija I., Tomljanović M., Novak Kujundžić R., Trošelj K.G. (2022). EZH2 inhibition and cisplatin as a combination anticancer therapy: An overview of preclinical studies. Cancers.

[21] Gounder M., Schoffski P., Jones R.L., Agulnik M., Cote G.M., Villalobos V.M., Attia S., Chugh R., Chen T.W.-W., Jahan T. (2020). Tazemetostat in advanced epitheliod sarcoma with loss of INI1/SMARCB1: An international, open-label phase 2 basket study. Lancet Oncol..

[22] Morschhauser F., Tilly H., Chaidos A., McKay P., Phillips T., Assouline S., Batlevi C.L., Campbell P., Ribrag V., Damaj G.L. (2020). Tazemetostat for patients with relapsed or refractory follicular lymphoma: An open-label, single-arm, multicentre, phase 2 trial. Lancet Oncol..

